# Cross-tissue and generation predictability of relative *Wolbachia* densities in the mosquito *Aedes aegypti*

**DOI:** 10.1186/s13071-022-05231-9

**Published:** 2022-04-12

**Authors:** Austin J. Mejia, H. L. C. Dutra, M. J. Jones, R. Perera, E. A. McGraw

**Affiliations:** 1 Department of Entomology, The Pennsylvania State University, University Park, PA 16802 USA; 2 The Center for Infectious Disease Dynamics, The Huck Institutes of the Life Sciences, The Pennsylvania State University, University Park, PA 16802 USA; 3 Department of Biology, The Pennsylvania State University, University Park, PA 16802 USA; 4 Center for Vector-Borne Infectious Diseases, Colorado State University, Fort Collins, CO 80523 USA

**Keywords:** *Wolbachia*, Density, *Aedes aegypti*, Mosquito, Symbiont

## Abstract

**Background:**

The insect endosymbiotic bacterium *Wolbachia* is being deployed in field populations of the mosquito *Aedes aegypti* for biological control. This microbe prevents the replication of human disease-causing viruses inside the vector, including dengue, Zika and chikungunya. Relative *Wolbachia* densities may in part predict the strength of this ‘viral blocking’ effect. Additionally, *Wolbachia* densities may affect the strength of the reproductive manipulations it induces, including cytoplasmic incompatibility (CI), maternal inheritance rates or induced fitness effects in the insect host. High rates of CI and maternal inheritance and low rates of fitness effects are also key to the successful spreading of *Wolbachia* through vector populations and its successful use in biocontrol. The factors that control *Wolbachia* densities are not completely understood.

**Methods:**

We used quantitative PCR-based methods to estimate relative density of the *Wolbachia w*AlbB strain in both the somatic and reproductive tissues of adult male and female mosquitoes, as well as in eggs. Using correlation analyses, we assessed whether densities in one tissue predict those in others within the same individual, but also across generations.

**Results:**

We found little relationship among the relative *Wolbachia* densities of different tissues in the same host. The results also show that there was very little relationship between *Wolbachia* densities in parents and those in offspring, both in the same and different tissues. The one exception was with ovary–egg relationships, where there was a strong positive association. Relative *Wolbachia* densities in reproductive tissues were always greater than those in the somatic tissues. Additionally, the densities were consistent in females over their lifetime regardless of tissue, whereas they were generally higher and more variable in males, particularly in the testes.

**Conclusions:**

Our results indicate that either stochastic processes or local tissue-based physiologies are more likely factors dictating *Wolbachia* densities in *Ae. aegypti* individuals, rather than shared embryonic environments or heritable genetic effects of the mosquito genome. These findings have implications for understanding how relative *Wolbachia* densities may evolve and/or be maintained over the long term in *Ae. aegypti*.

**Graphical Abstract:**

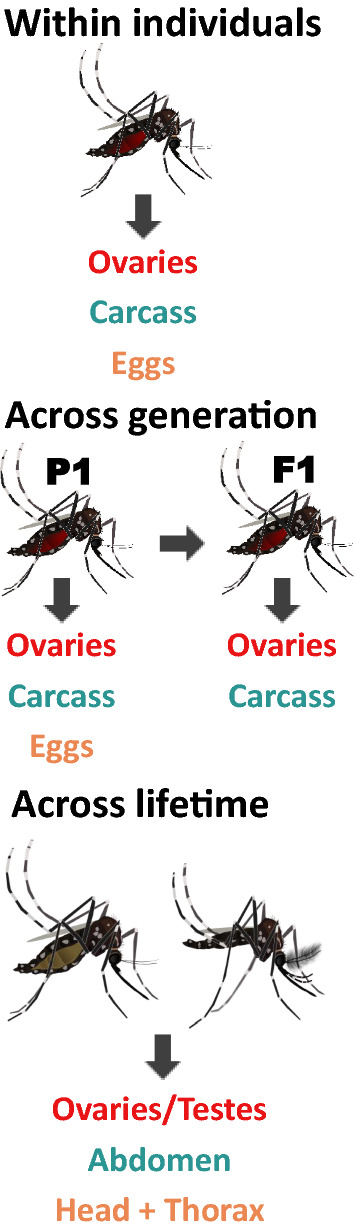

**Supplementary Information:**

The online version contains supplementary material available at 10.1186/s13071-022-05231-9.

## Background

The global geographic range of the mosquito vector *Aedes aegypti*, which transmits the human disease-causing viruses dengue, Zika, chikungunya and yellow fever [1], is expanding [2]. Because there are no viable vaccines for these viruses, vector control remains the primary mechanism for limiting human disease [3]. *Wolbachia pipientis* is an endosymbiotic bacterium found in approximately 50% of all known insect species [4]. The symbiont induces two phenotypes that complement one another, which can be used in vector-borne disease control. First, it causes cytoplasmic incompatibility (CI), whereby offspring from crosses between *Wolbachia*-infected males and *Wolbachia*-free females are non-viable. The result is that *Wolbachia*-infected females have greater relative reproductive success and because *Wolbachia* is maternally inherited vertically via the egg, the symbiont spreads through populations [5]. Second, *Wolbachia* has also been found to limit the replication of co-infecting viruses in many insects, including *Ae. aegypti* [6–8], in a trait known as *Wolbachia*-mediated pathogen blocking (WMPB). *Aedes aegypti* in the wild are naturally free of *Wolbachia*, but laboratory populations have been artificially and stably infected with the symbiont [9–13].

In the field, *Wolbachia* is being evaluated for vector-borne disease control through two strategies: population suppression and population replacement [14]. Suppression involves releasing *Wolbachia*-infected males only, with the aim to prevent the successful reproduction of wild *Wolbachia*-free females, leading to population reductions. In replacement strategies, *Wolbachia*-infected females are released in large numbers to seed the next generation with *Wolbachia-*infected offspring. The daughters become part of the maternal transmission cycle and the sons assist with *Wolbachia* spread via the action of CI. The result is a population with high rates of *Wolbachia* infection and a poor ability to transmit viruses [15, 16]. Both strategies are showing high rates of efficacy in the field [17, 18]. The continued success of these approaches relies on the ongoing strength of CI and WMPB expression. Studies appear to show that *Wolbachia* densities predict the strengths of both CI and WMPB [19–25].

The factors that control *Wolbachia* densities are not fully understood [26], but appear to involve both host and symbiont genetics [27, 28] and a range of environmental effects, including temperature and host nutrition [28–31]. Even within an individual insect, *Wolbachia* densities can vary highly between tissues, with reproductive tissues often exhibiting higher densities, although in Drosophila this can depend on the *Wolbachia* strain [30, 32]. Higher *Wolbachia* densities in reproductive tissues have been noted in the mosquitoes *Ae. aegypti* [33], *Ae. albopictus* [34] and *Culex pipiens* [35]*.* The relative contributions of *Wolbachia*/vector genetics versus environmental effects in determining *Wolbachia* tissue densities is not known. Studying the heritability of *Wolbachia* densities in female lineages is challenging, given that the shared environment of the ovaries/eggs confounds any estimates of contributions from genetic factors. In this study, we sought to understand the relationship between *Wolbachia* densities in somatic and reproductive tissues within individuals and across generations in the artificially *w*AlbB strain-transinfected *Ae. aegypti* using quantitative PCR-based methods. An understanding of the relative role of genes and environment in determining *Wolbachia* densities may have consequences for the deployment and use of *Wolbachia*-based biocontrol where key phenotypes depend on density.

## Methods

### *Aedes aegypti* rearing

We used a population of *Ae. aegypti* infected with the *w*AlbB [11] strain of *Wolbachia* (kind gift from Zhiyong Xi, Michigan State University). Prior to experimentation, we outcrossed* w*AlbB-infected female mosquitoes to *Wolbachia*-free male mosquitoes recently obtained from Monterrey, Mexico (Pablo Manrique-Saide, Universidad Autónama de Yucatán) for three generations to increase genetic diversity. Eggs were hatched in 40 × 30 × 8-cm plastic trays containing 2 l of distilled deoxygenated water. Larvae were maintained at a density of approximately 250 per tray and fed Tetramin fish food (Tetra GmbH, Melle, Germany) ad libitum. Pupae were collected in plastic cups and placed in 45-cm square breeding cages (BioQuip Products, Rancho Dominguez, CA, USA) in populations of approximately 300 individuals. Adult mosquitoes were fed a 10% sucrose solution ad libitum. Mosquitoes were blood-fed when 9–11 days of age with human blood using a Hemotek feeder (Hemotek Ltd., Blackburn, UK) warmed to 37 °C. For experiments that involved egg collection, 3 days post blood-feeding, females were individually placed in 70-ml oviposition cups containing moist filter paper, with access to 10% sucrose.

### Experimental design

**Fig. 1:**
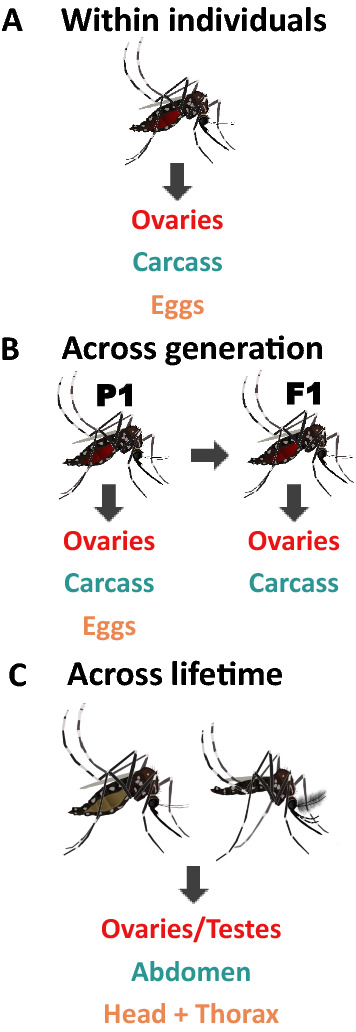
Tissues collected for each experiment. **a** Within-individual tissue correlation, **b** parent–offspring (P_1_–F_1_) correlation, **c** Tissue comparisons over the mosquito’s lifespan

*Wolbachia* densities were measured using three approaches: (i) between somatic and reproductive tissues in the same individuals; (ii) across generations in tissues of parents and offspring; and (iii) in tissues of the same individuals across their lifespan (Fig. 1). Our aim was to examine whether relative tissue densities correlated in these different contexts. For the within-individual mosquito comparisons (Fig. 1a), we set up two experiments. First, we set up 206 mated blood-fed individuals as isofemales at 9–11 days of age. We dissected the ovaries and the carcass (remaining tissues) at 15–17 days of age, or at approximately 6 days post-feeding. Second, we set up two replicate groups of 18 blood-feed isofemales so that in addition to ovaries and the carcass we could also correlate egg densities within individuals. Eggs were extracted for DNA analysis in groups of 10 per isofemale, after pilot experiments revealed this was the minimum pool size needed for consistent *Wolbachia* density estimation. For across-generation comparisons (Fig. 1b), we also set up blood-fed isofemales (~ 200) in the same manner as described above. After collecting their eggs, we dissected ovaries and the carcass in the mothers (P_1_). We hatched the eggs laid by each isofemale separately, and then reared, fed and dissected these F_1_ families as described for P_1_. *Wolbachia* densities were estimated from tissues extracted individually from two to three F_1_ daughters per P_1_ mother and then averaged. We also wanted to correlate relative *Wolbachia* density in the eggs to *Wolbachia* density in the ovaries and the carcass across a generation. Therefore, we set up a new colony, collected eggs from P_1_ mothers and extracted pools of 10 eggs per female. We hatched the remaining eggs to create 20 cages of approximately 50 individuals. F_1_ families were dissected identically as described above for F_1_ families. Relative *Wolbachia* densities were estimated in tissues extracted from three to eight F_1_ daughters per P_1_ mother and then averaged. For the experiment examining relative *Wolbachia* densities in tissue over the mosquito’s lifespan (Fig. 1c) we set up a cage of approximately 250 individuals, and then dissected males and females not blood-fed for their reproductive tissues, abdomen, head and thorax at 5, 10 and 15 days of adulthood.

### Dissections and DNA extraction

Females were anesthetized using CO_2_ and dissected in 1× phosphate buffered saline (PBS). Tissues were collected and placed in a 2-ml tube containing 50 μl of PBS and a 3-mm glass bead. Dissected tissues were stored at − 80 °C until processing. Similarly, eggs were collected in groups of 10. To extract DNA from the eggs, tubes containing eggs were filled with 50 μl of extraction buffer (10 mM Tris buffer, 1 mM EDTA, 50 mM NaCl, and proteinase K). The samples were then homogenized with a bead ruptor (OMNI International, Kennesaw, GA, USA) for 90 s, centrifuged at 2000 *g* for 2 min and then incubated at 56 °C for 5 min and at 98 °C for 5 min. A final centrifugation step was performed at 2000 *g* for 2 min to pellet any remaining mosquito tissue. Samples were diluted 1:10 using DNAse/RNAse-free water prior to quantification.

### *Wolbachia* quantification

While there are methods for estimating absolute numbers of *Wolbachia* [36], we chose to measure relative abundance. This method is more appropriate when the aim is to capture *Wolbachia* density in a given tissue and to compare *Wolbachia* density across tissues where the size of tissue (number [*n*] cells) will vary. Densities rather than absolute numbers may be more revealing when attempting to capture the ‘concentration’ of *Wolbachia*, which may affect the strength of *Wolbachi*a-mediated phenotypes [19–25]. The relative method of estimation could be misleading, however, if ploidy numbers differ across tissues [36]. Since ploidy by cell or tissue type has scarcely been studied in mosquitoes, in addition to providing *Wolbachia* gene to host gene ratios, we also provide our raw crossing point (CP) values for the mosquito control gene in the Additional files to demonstrate their uniformity. Average *rps17* values for all tissues were found to vary by less than twofold across the samples (see specific figures/Additional files in Results section), suggesting low variability. Relative *Wolbachia* densities were quantified by real-time PCR (qPCR) using Livak’s method [37] and a set of previously published primers for the *w*AlbB* ankyrin repeat domain* gene [38] and the mosquito ribosomal subunit protein S17 (RpS17) [39]. The *Wolbachia* primers were *w*AlbB_F (5′-CCTTACCTCCTGCACAACAA) and *w*AlbB_R (5′-GGATTGTCCAGTGGCCTTA) [38], and the mosquito primers were *RPS17*_F (5′-TCCGTGGTATCTCCATCAAGCT) and *RPS17*_R (5′-CACTTCCGGCACGTAGTTGTC) [39]. qPCR was carried out on a LightCycler 480 Real-Time PCR System (Roche, Basel, Switzerland), using the equation $\frac{{2^{{ - {\text{wAlbB}}}} }}{{2^{{ - {\text{RPS}}17}} }}$ [37], in a total reaction volume of 10 μl (5 μl of 2× PerfeCTa SYBR Green SuperMix [Quantabio, Beverly, MA, USA], 0.2 μl of each forward and reverse primers [10 μM], 2.6 μl of nuclease-free water, 8 μl template DNA). The qPCR cycling profile was: denaturation at 95 °C for 5 min; 45 cycles of 95 °C for 10 s, 60 °C for 15 s and extension at 72 °C for 10 s; followed by a melt curve analysis.

### Statistical analysis

All statistical analyses for the ‘within-individual’ (Fig. 1a) and ‘across-generation’ (Fig. 1b) experiments were performed using GraphPad Prism version 9.1.0 for Windows (GraphPad Software, San Diego, CA, USA). Data were checked for normality before performing the analysis and logarithmically transformed when necessary. All relative densities when depicted in scatter plots were plotted on a log axis; fitted regression lines, although linear, can therefore appear curved. Paired t-tests were performed when comparing ovaries and carcass. A one-way analysis of variance (ANOVA) was used to compare relative *Wolbachia* densities with ‘Tissue’ as a fixed effect. Tukey’s post hoc comparisons were used to individually compare the densities in the ovary, carcass and eggs. Analysis of *Wolbachia* densities in the ‘across-mosquito lifespan’ experiment (Fig. 1c) was performed in JMP 16.0.0 (SAS Institute Inc., Cary, NC, USA). A three-way ANOVA was used to compare the factors sex, time and tissue, followed by selected post hoc comparisons.

## Results

### Within-individual relative *Wolbachia* tissue density comparisons

**Fig. 2:**
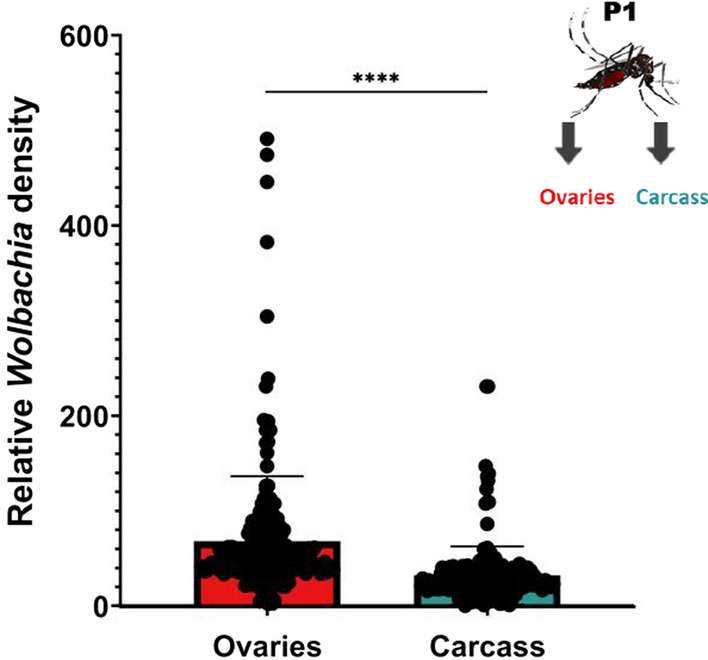
Relative *Wolbachia* densities (*ankyrin repeat domain* to rps17) in the ovaries and the carcass of *Aedes aegypti* in the same generation of mothers (P_1_)(*n* = 206; *P* < 0.0001, paired t-test). Bars indicate tissue means ± standard error (SE). Asterisks indicate significant difference at ****P ≤ 0.0001

**Fig. 3:**
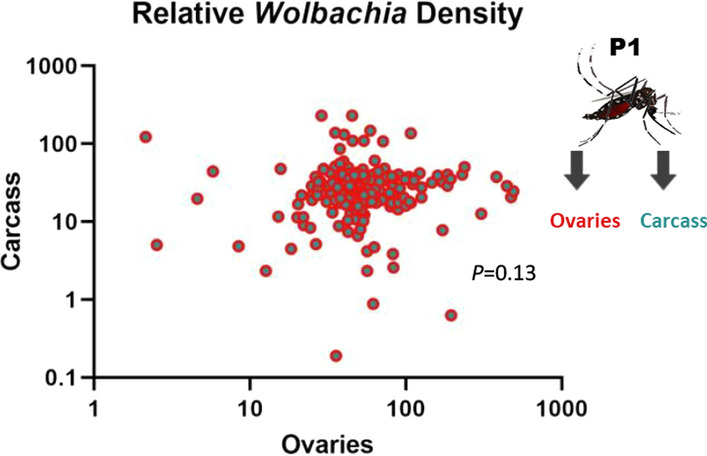
Relationship between relative *Wolbachia* densities (*ankyrin repeat domain* to *rps17*) in the ovaries and the carcass of *Ae. aegypti* in the same generation of mothers (P_1_). *n* = 206

**Fig. 4:**
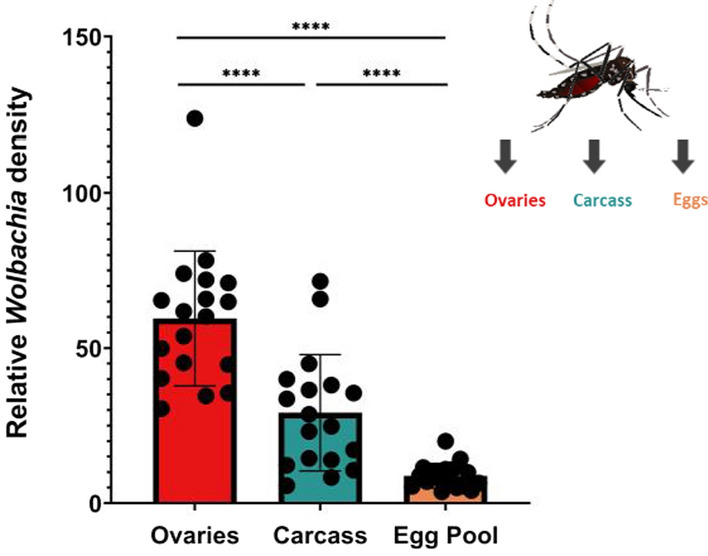
Relative *Wolbachia* densities (*ankyrin repeat domain* to *rps17*) in the ovaries, carcass and eggs of *Ae. aegypti.* One-way analysis of variance *P* < 0.0001, post-hoc Tukey’s test: ovaries vs eggs: *P* < 0.0001; carcass vs eggs: *P* < 0.0001; ovaries vs carcass: *P* < 0.0001. *n* = 18 individuals. Bars indicate tissue means ± SE. Asterisks indicate significant difference at ****P ≤ 0.0001

**Fig. 5:**
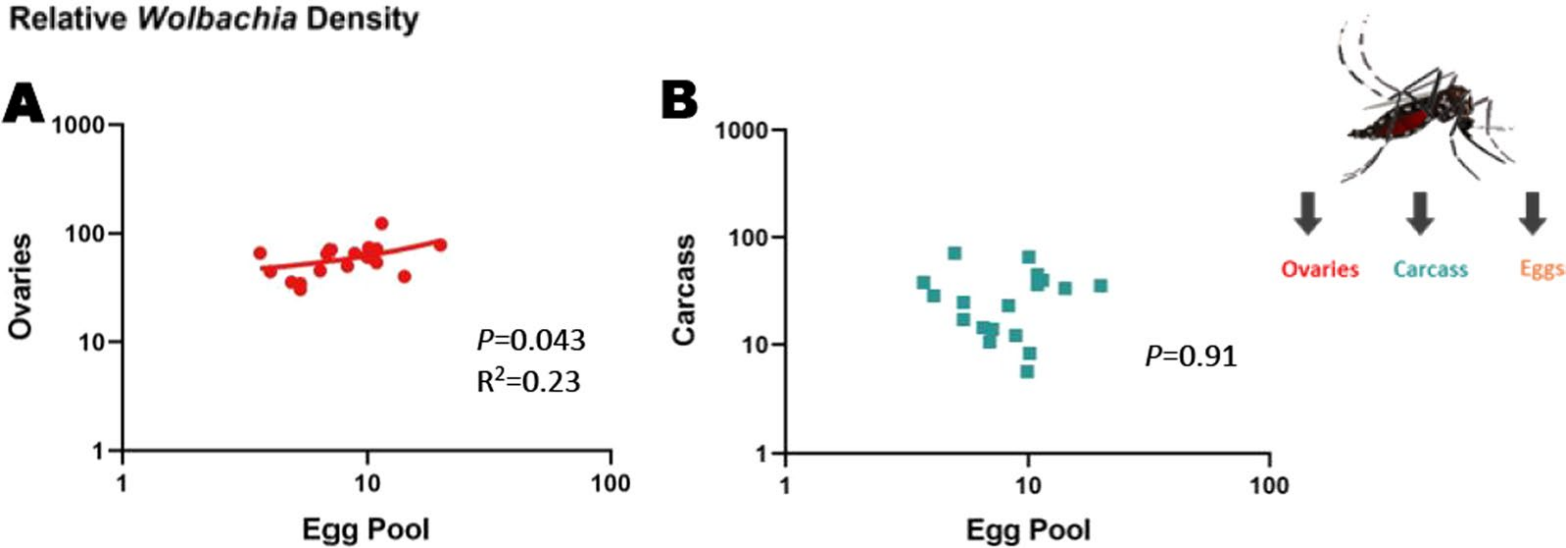
Relative *Wolbachia* densities (*ankyrin repeat domain* to *rps17*) in the ovaries, carcass and eggs of replicate group 1 of *Ae. aegypti.* **a** *Wolbachia* densities in the eggs vs the ovaries of *Ae. aegypti* in replicate group 1, **b** *Wolbachia* densities in the eggs versus the carcass of *Ae. aegypti* in replicate group 1. *n* = 18 individuals per group

Relative *Wolbachia* density between ovaries and carcass was measured at approximately 6 days after blood-feeding (15–17 days of adulthood) in the same individuals to see whether tissue densities were correlated with one another (Fig. 1a). We found that *Wolbachia* densities were significantly greater in the ovaries (twofold higher) than in the carcass (*P* < 0.0001) (Fig. 2; Additional file 1: Figure S1 [raw CP data]). Relative densities ranged from ~ 2 to ~ 491 in the ovaries and from ~ 0 to ~ 230 in the carcass; these values also reflect a wider variation in density in the reproductive tissue. We found no correlation between the relative *Wolbachia* densities of the ovaries and carcass (*P* = 0.13) (Fig. 3). We then measured relative *Wolbachia* densities between the ovaries, carcass and eggs in the same individuals to assess whether there were any correlations (Fig. 1a). To accomplish this, we set up two replicate groups of 18 individuals each. We found that *Wolbachia* densities in the ovaries were fivefold (Fig. 4; Additional file 2: Figure S2 [raw CP data]) and tenfold (Additional file 3: Fig. S3) higher, respectively, than those in eggs produced by the same individuals (Tukey’s multiple comparison test: *P* ≤ 0.0001). *Wolbachia* densities in the carcass were also twofold (Fig. 4) (Tukey’s multiple comparison test: *P* ≤ 0.0001) and fourfold (Additional file 3: Figure S3) (Tukey’s multiple comparison test: *P* ≤ 0.0001) higher than those in the eggs. In one group, *Wolbachia* densities in the ovaries were twofold higher than those in the carcass (Fig. 4) (Tukey’s multiple comparison test: *P* ≤ 0.0001), but there was no significant difference in the second replicate (Additional file 3: Figure S3) (Tukey’s multiple comparison test: *P* = 0.33). Both groups exhibited the same trend of higher relative *Wolbachia* density in eggs correlating with higher *Wolbachia* density in the ovaries for replicate 1 (*P* = 0.043) (Fig. 5a) and replicate 2 (*P* = 0.0062) (Additional file 4: Figure. S4A). In both replicate 1 (*P* = 0.91) (Fig. 5b) and replicate 2 (*P* = 0.13) (Additional file 4: Figure S4B) there was no correlation between *Wolbachia* densities in the egg and carcass. Overall, *Wolbachia* densities in eggs were far less variable than those in the ovaries or carcass.

### Across-generation relative *Wolbachia* tissue density comparisons

**Fig. 6:**
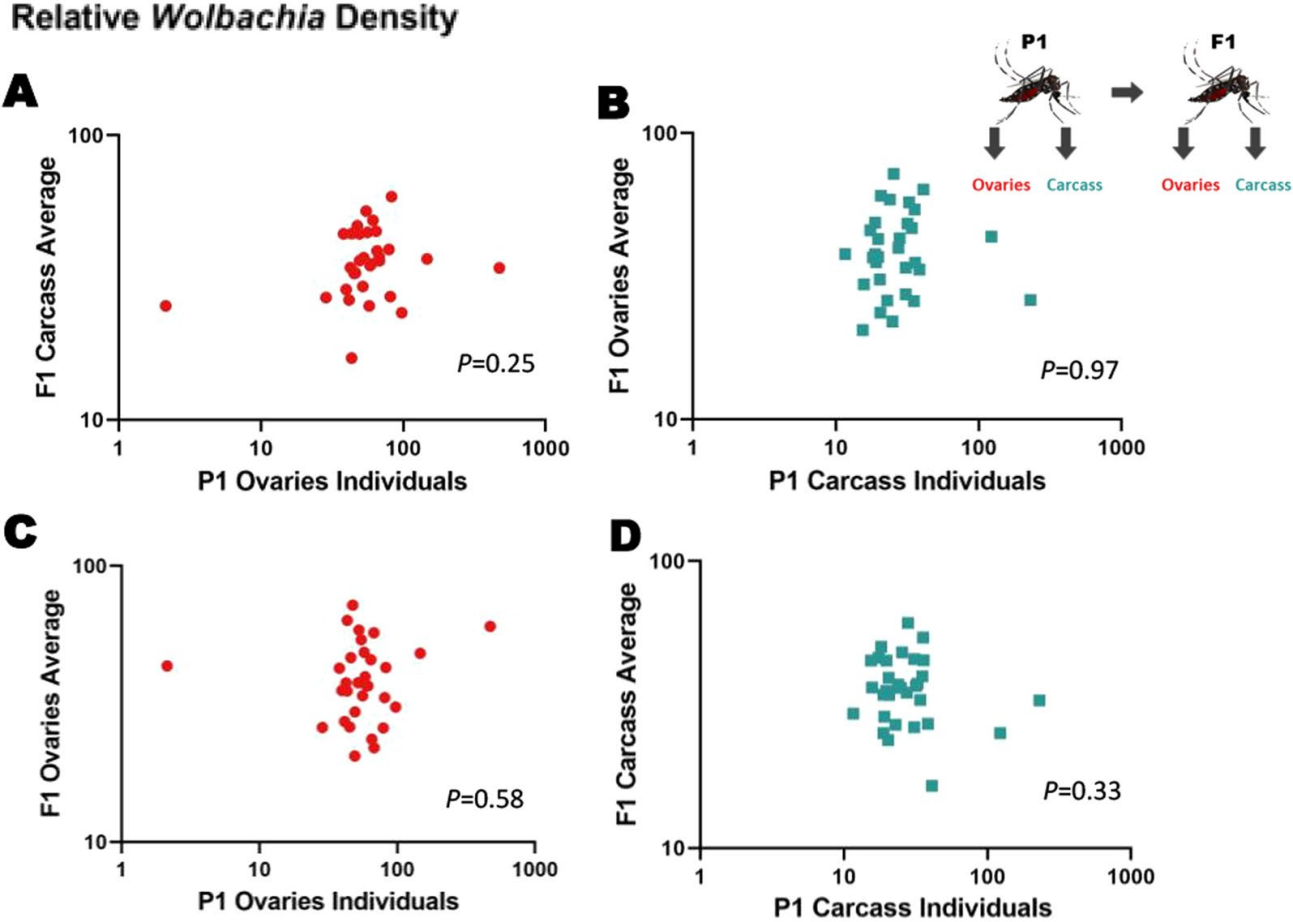
Relative *Wolbachia* densities (*ankyrin repeat domain* to *rps17*) in the tissues of mothers (P1) vs daughters (F1). **a** *Wolbachia* densities in the ovaries of P1 vs carcass of F_1_ in *Ae. aegypti*. **b** *Wolbachia* densities in the carcass of P1 vs the ovaries of F1 in *Ae. aegypti*. **c** *Wolbachia* densities in the ovaries of P1 versus the ovaries of F1 in *Ae. aegypti*. **d** *Wolbachia* densities in the carcass of P1 vs the carcass of daughters F1 in *Ae. aegypti*. Each data point represents the average of 2–3 individuals.* n* = 31 data points in** a**,** b**; *n* = 30 data points in** c**,** d**

**Fig. 7:**
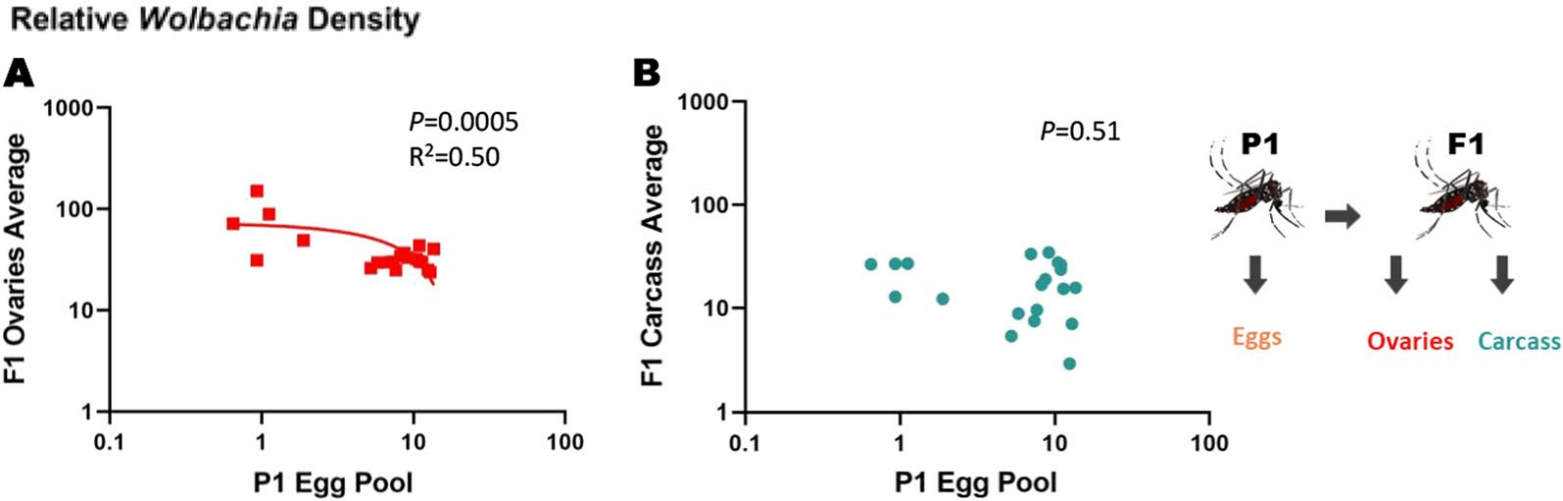
Relative *Wolbachia* densities (*ankyrin repeat domain* to *rps17*) in the eggs of mothers (P_1_) vs tissues of daughters (F_1_). **a** *Wolbachia* densities in the eggs vs the ovaries of *Ae. aegypti,* **b** *Wolbachia* densities in the eggs vs the carcass of *Ae. aegypti.* Each data point represents the average of 3–8 individuals. *n* = 20 data points

We then examined whether tissue densities in female offspring could be predicted based on densities in the female parent (Fig. 1b). We saw no relationship between *Wolbachia* densities in P_1_ ovaries and F_1_ carcass (*P* = 0.25) (Fig. 6a; Additional file 5: Figure S5 [raw CP data]), nor between *Wolbachia* densities in P_1_ carcass and F_1_ ovaries (*P* = 0.97) (Fig. 6b). Similarly, we found no correlation between *Wolbachia* densities in P_1_ and F_1_ ovaries (*P* = 0.58) (Fig. 6c), nor between *Wolbachia* densities in P_1_ and F_1_ carcass (*P* = 0.33) (Fig. 6d). A negative correlation was found between *Wolbachia* densities in P_1_ eggs and F_1_ ovaries (*P* = 0.0005) (Fig. 7a; Additional file 6: Figure. S6 [raw CP data]). No correlation was found between *Wolbachia* densities in P_1_ eggs and F_1_ carcass (*P* = 0.51) (Fig. 7b).

### Across-lifetime relative *Wolbachia* tissue density comparisons

**Fig. 8:**
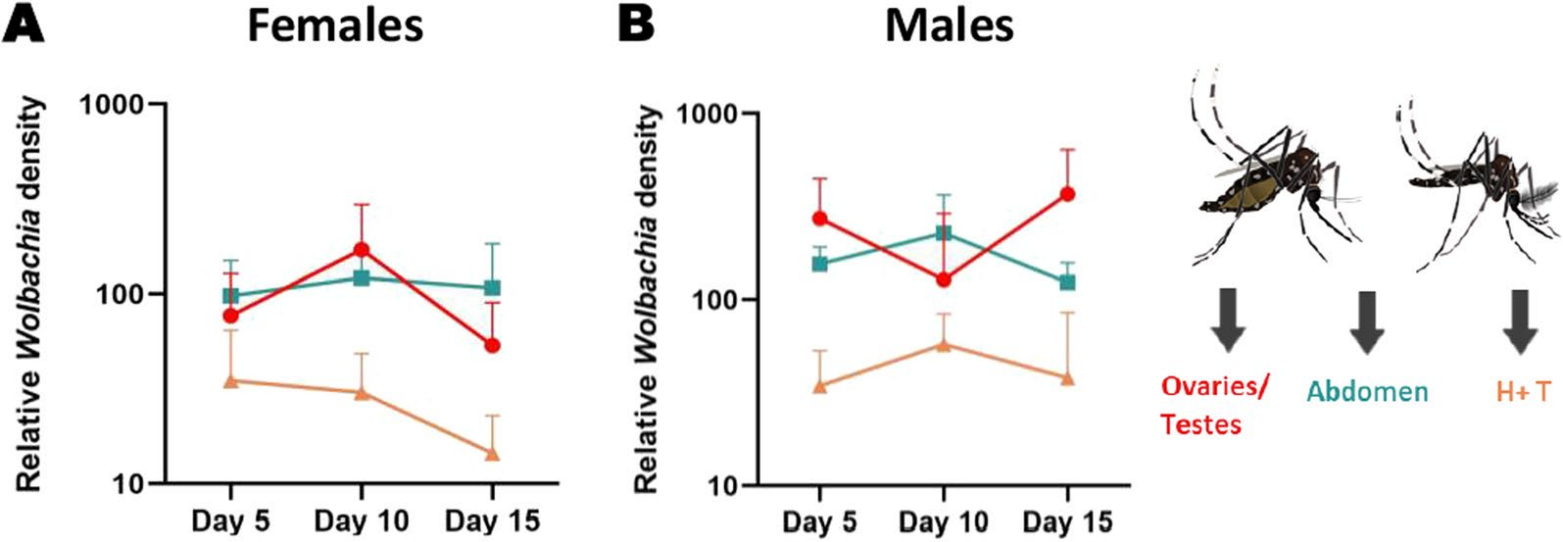
Relative *Wolbachia* densities (*ankyrin repeat domain* to *rps17*) across *Ae. aegypti* lifetime. **a** Relationship between *Wolbachia* densities in the ovaries, abdomen and head + thorax (H + T) of *Ae. aegypti* females at different time points. *n* = 14–15 individuals. (Tissue = *P* ≤ 0.0001; Time = *P* = 0.50; Time × Tissue = *P* = 0.60). **b** Relationship between *Wolbachia* densities in the testes, abdomen and H + T of *Ae. aegypti* males at different time points. *n* = 12–15 individuals. Only sex (*P* < 0.001) and tissue were significant (*P* < 0.001)

To assess whether symbiont densities change with time, relative *Wolbachia* density was measured in the reproductive tissue, the abdomen and a combination of the head and thorax (H + T) of male and non-blood-fed female mosquitoes at 5, 10, and 15 days of age. A three-way ANOVA between sex, time and tissue resulted in sex (*P* < 0.001) and tissue (*P* < 0.001) being significant, as well as the interaction between these two factors (*P* = 0.0004) (Fig. 8). *Wolbachia* densities in the H + T (Tukey’s multiple comparison test: *P* = 0.0098) and abdomen (Tukey’s multiple comparison test: *P* = 0.0007) remained largely stable over the lifetime in both males and females. On average across all days, *Wolbachia* densities in the reproductive tissue in males were more variable over time and higher than those in female reproductive tissue (Tukey’s multiple comparison test: *P* < 0.0001).

## Discussion

The distribution of *Wolbachia* in somatic tissues varies between species, but the symbiont can be found in the head, muscles, midgut, salivary gland, fat body, and reproductive tissues [40–42]. In keeping with previous findings for *Drosophila* [43] and *Aedes* [44], the relative *Wolbachia* densities reported in our study were higher in reproductive tissues compared to somatic tissues. Additionally, we found that *Wolbachia* densities in the ovaries and carcass, and those in the carcass and eggs, in the same individual, are largely independent of one another. One of the primary drivers of this disconnect could be the distribution of *Wolbachia* in the early embryo. In *Drosophila*, *Wolbachia* attach to the proliferating nuclei and use this relationship to hitchhike through the oocyte [45]. This places some *Wolbachia* in the periphery of the egg, where reproductive cells are formed [46]. However, a high fraction of the bacteria remains throughout the oocyte [47]. It is believed that this early embryonic distribution is what dictates which somatic tissues will have *Wolbachia* and partially explains their relative *Wolbachia* densities [41]. Little is known about *Wolbachia’s* life-cycle during the embryonic development of mosquitoes. However, *Drosophila* and *Aedes* have very similar embryonic developmental stages that differentiate by timing [48].

Local tissue-specific factors could also be driving differences in relative *Wolbachia* densities, such as immunity, physical niches or access to nutritional resources. For example, the insect immune response can positively or negatively affect *Wolbachia* densities, in a tissue-specific manner. Autophagy is a pathway that involves the degradation of “unwanted” components, such as pathogenic bacteria. In the somatic cells of male *Drosophila*, the autophagic response reduces *Wolbachia* density, but the opposite occurs in germ cells of females [49]. In multiple studies, infection with another bacterium [50] or virus [51, 52] that triggers the innate Toll and immune deficiency (IMD) pathways appears to also cause reductions in *Wolbachia* density*.* We know from transcriptional studies that the activity of these pathways varies across mosquito tissues, including the midgut, carcass and salivary gland, when induced by infection [53], but their basal expression can also vary as per an examination of the control data for these same studies [54, 55]. One somatic tissue with a very high *Wolbachia* relative density in *Ae. aegypti* is the Malpighian Tubules [40]. These tissues are the main site of nitrogen secretion and as such they may especially facilitate *Wolbachia* growth, given that the symbiont primarily consumes host nucleotides [56], a large source of nitrogen. This same study also revealed pockets of cells within particular tissues, such as the midgut epithelia, thoracic ganglia and the salivary glands, that exhibit higher relative densities than the surrounding tissues [40]. The reason for either *Wolbachia’s* tropism to particular cell types or greater replicative success in these sub-tissue level environments is unknown.

Our results did show a correlation between relative *Wolbachia* densities in the ovaries and eggs produced from the same individual, which is expected given the egg’s origin and *Wolbachia’s* vertical inheritance [57]. The ongoing success of *Wolbachia’s* transmission depends on its density in the ovaries. A range of studies on Drosophila shed light on the interactions between *Wolbachia* and the female germline that may also be relevant for mosquitoes. For example, *Wolbachia* increases the production of fly proteins in the ovaries that protect the germline from iron toxicity and oxidative stress, and increase the rate of stem-cell division [58]. The increased prevalence of these proteins may aid *Wolbachia’s* own proliferation and ensure transmission [58]. Also, *Wolbachia* has a tropism for the ovarian stem-cell niche. Once there, *Wolbachia* increases germline stem-cell division and stops programmed cell death, resulting in higher egg production [59]. Additionally, *Wolbachia’s* tropism to ovarian stem-cell niches has been found to increase bacterial density in the germline [60]. Therefore, *Wolbachia* ensures vertical inheritance by increasing egg production and its own density in the germline.

In contrast to the ovary/egg relationship, we did not see predictability of relative *Wolbachia* densities across generations for other tissues. In our comparison of *Wolbachia *density across generations, we considered the relative contributions of genes and environment to the determination of density versus stochastic processes. Temperature and diet have been shown to affect relative *Wolbachia* densities [28, 29, 61, 62]. However, a previous study showed that *w*AlbB remains at a constant density between 26 °C and 37 °C [63], and under laboratory rearing conditions our temperatures should be largely constant. Similarly, given the low-density rearing of larvae and ad libitum food delivery in both juveniles and adults, nutrition should have minimal impact on densities in our study design. Host genetic factors cause varying *Wolbachia* density in arthropods [26, 30]. Our poor cross-generation predictability, however, is more in keeping with the results from a recent study demonstrating that genetic drift is a more likely dictator of density [64]. The cause of this drift can likely be attributed to the uneven passage of *Wolbachia* from mother to egg, causing siblings to have varying densities [64].

One caveat to our study is that we focused only on the *w*AlbB *Wolbachia* strain. Future studies may wish to assess the generality of our findings for other strains in *Ae. aegypti*. While a previous study in *Ae. albopictus* [9] also showed no relationship between mother to offspring densities for both the *w*AlbA and *w*AlbB strains, the more distantly related *w*Mel strain could differ. A recent study in flies showed very large differences in relative *Wolbachia* tissue densities depending on the *Wolbachia* strain:host species pairing [30]. Our findings have potential implications for *Wolbachia*-based biocontrol in the field. In the longer term, any directional selection on *Wolbachia* densities in the ovaries may not have a similar predictable effect on the body-wide densities, as well as the converse. This is important as the former is thought to maintain transmission and CI expression [65, 66], whereas the latter is likely to control pathogen blocking [67]. Infection of both types of tissues may have direct impacts on host fitness [68]. This also means that artificial selection to create mosquito lines with higher or lower *Wolbachia* densities in their various tissues is unlikely to be effective. Identifying *Wolbachi*a strains for transinfection that exhibit differences in density either singly or when in superinfection with other strains [33, 40, 69] may offer the most effective means for generating strain density diversity [70].

## Conclusions

The results of this study suggest that, in *Ae. aegypti*, local tissue-based environments (e.g. nutrition, cellular niches, immunity), initial differential distributions of *Wolbachia* in the dividing embryo or stochastic factors (e.g. partitioning of density-associated *Wolbachia* genotypes in the embryo) are likely to be more powerful determinants of relative symbiont densities than shared embryonic environments and shared inheritance through a female genetic line. Our finding of a relatively narrow variation in *Wolbachia* densities in eggs, ultimately resulting in highly variable densities in adult tissues, is also in keeping with this hypothesis. Future comparative studies may seek to understand how distinct tissue and cellular niches either promote or limit relative *Wolbachia* densities. The growing use of single-cell RNAseq approaches in insects [71] may assist with these comparisons. At the level of the vector, the effect of environmental conditions, more representative of natural field settings [72], may introduce further variability in densities, *Wolbachia* inheritance and the expression of *Wolbachia*-induced traits that are key for biocontrol strategies.

## Supplementary Information

**Additional file 1: Figure S1.** Crossing point values of *rps17* in the ovaries and the carcass of *Ae. aegypti* in the same generation of mothers (P1). These data pertain to Fig. 2 in the main text. *n* = 206, *P* < 0.0001 (paired t-test). Bars indicate tissue means ± SE; ****Significant difference at *P* ≤ 0.0001.

**Additional file 2: Figure S2.** Crossing point values of *rps17* in the ovaries, carcass and eggs of *Ae. aegypti.* These data pertain to Fig. 4 in the main text. One-way ANOVA *P* < 0.0001; post-hoc Tukey’s test: ovaries vs eggs: *P* < 0.0001; carcass vs eggs: *P* < 0.0001; ovaries vs carcass: *P* < 0.0001. *n* = 18 individuals. Bars indicate tissue means ± SE. ****Significant difference at *P* ≤ 0.0001.

**Additional file 3: Figure S3.** Relative *Wolbachia* densities (*ankyrin repeat domain* to *rps17*) in the ovaries, carcass and eggs of *Ae. aegypti* in replicate group 2. One-way ANOVA *P* < 0.0001; post-hoc Tukey’s test: ovaries vs eggs: *P* < 0.0001; carcass vs eggs: *P* < 0.0001; ovaries vs carcass: *P* = 0.33. *n* = 18 individuals. Bars indicate tissue means ± SE. ns, not significant; asterisks indicate significant difference at ***P ≤ 0.001 and ****P ≤ 0.0001, respectively.

**Additional file 4: Figure S4.** Relative *Wolbachia* densities (*ankyrin repeat domain* to *rps17*) in the ovaries, carcass and eggs of *Ae. aegypti* in replicate group 2*.*** A** *Wolbachia* densities in the eggs vs the ovaries of *Ae. aegypti* in replicate group 2. B. *Wolbachia* densities in the eggs vs the carcass of *Ae. aegypti* in replicate group 2. *n* = 18 individuals.

**Additional file 5: Figure S5.** Crossing point values of *rps17* in the tissues of mothers (P_1_) and daughters (F_1_). These data pertain to Fig. 6 in the main text. **A** Crossing point values of *rps17* of P1 in *Ae. aegypti*. **B** Crossing point values of *rps17* of F1 in *Ae. aegypti.* Figure A has *n* = 31 individuals while B has *n* = 78 individuals. Bars indicate tissue means ± SE; ****P ≤ 0.0001.

**Additional file 6: Figure S6.** Crossing point values of *rps17* in the eggs of mothers (P1) and tissues of daughters (F1). These data pertain to Fig. 7 in the main text. For P1 eggs *n* = 20 individuals, while for ovaries and carcass *n* = 94 individuals. Bars indicate tissue means ± SE. ns, Not significant; asterisks indicate significant difference at ****P ≤ 0.0001.

**Additional file 7: Figure S7.** Relative *Wolbachia* densities (*ankyrin repeat domain* to *rps17*) in the ovaries and the carcass of *Ae. aegypti* in the same generation of daughters (F1). *n* = 79, *P* = 0.21 (paired t-test). Bars indicate tissue means ± SE. ns, Not significant.

**Additional file 8: Figure S8.** Relationship between relative *Wolbachia* densities in the ovaries and the carcass of *Ae. aegypti* in the same generation of daughters (F1). *n* = 79.

## Data Availability

All raw data for the study can be found upon publication in figshare 10.6084/m9.figshare.15129894.

## Abbreviations

CI: Cytoplasmic incompatibility
CP: Crossing point
H + T: Head and thorax
WMPB: *Wolbachia*-Mediated Pathogen Blocking
