# Supplementary figures and images for: Cross-tissue and generation predictability of relative Wolbachia densities in the mosquito Aedes aegypti
Source: Parasit Vectors. 2022 Apr 12;15:128. doi: 10.1186/s13071-022-05231-9 (PMC9004076; doi:10.1186/s13071-022-05231-9)

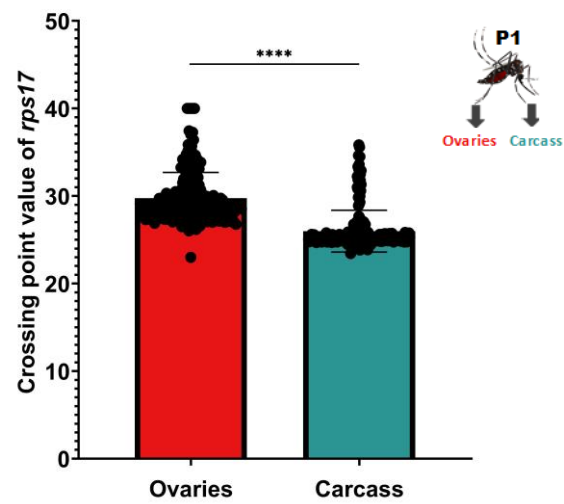

Supplement: Supplementary file 1 — Additional file 1: Figure S1. Crossing point values of rps17 in the ovaries and the carcass of Ae. aegypti in the same generation of mothers (P1). These data pertain to Fig. 2 in the main text. n = 206, P < 0.0001 (paired t-test). Bars indicate tissue means ± SE; ****Significant difference at P ≤ 0.0001. [file 13071_2022_5231_MOESM1_ESM.pdf]

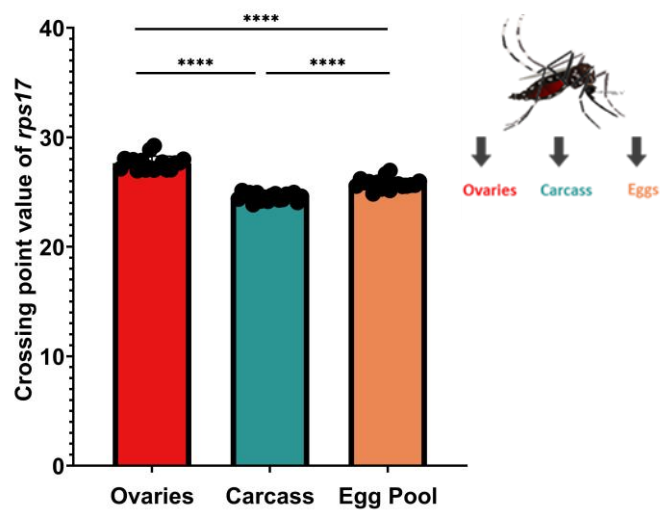

Supplement: Supplementary file 2 — Additional file 2: Figure S2. Crossing point values of rps17 in the ovaries, carcass and eggs of Ae. aegypti. These data pertain to Fig. 4 in the main text. One-way ANOVA P < 0.0001; post-hoc Tukey’s test: ovaries vs eggs: P < 0.0001; carcass vs eggs: P < 0.0001; ovaries vs carcass: P < 0.0001. n = 18 individuals. Bars indicate tissue means ± SE. ****Significant difference at P ≤ 0.0001. [file 13071_2022_5231_MOESM2_ESM.pdf]

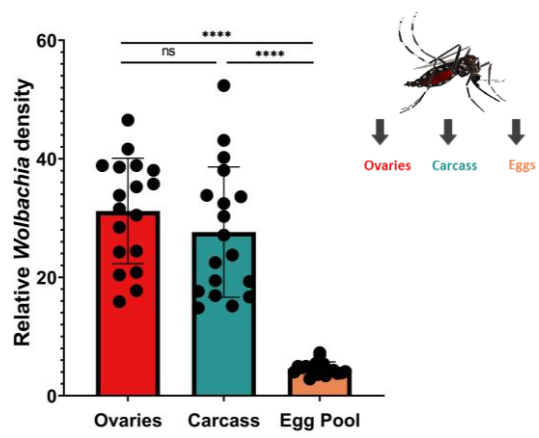

Supplement: Supplementary file 3 — Additional file 3: Figure S3. Relative Wolbachia densities (ankyrin repeat domain to rps17) in the ovaries, carcass and eggs of Ae. aegypti in replicate group 2. One-way ANOVA P < 0.0001; post-hoc Tukey’s test: ovaries vs eggs: P < 0.0001; carcass vs eggs: P < 0.0001; ovaries vs carcass: P = 0.33. n = 18 individuals. Bars indicate tissue means ± SE. ns, not significant; asterisks indicate significant difference at ***P ≤ 0.001 and ****P ≤ 0.0001, respectively. [file 13071_2022_5231_MOESM3_ESM.pdf]

Relative *Wolbachia* Density

**A**

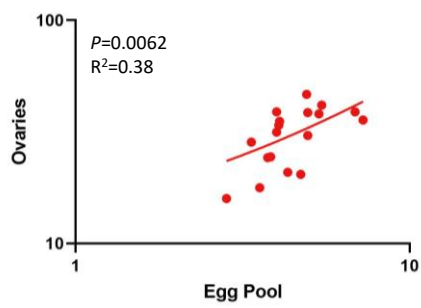

**B**

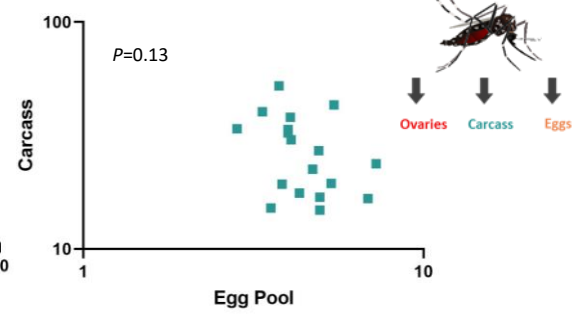

Supplement: Supplementary file 4 — Additional file 4: Figure S4. Relative Wolbachia densities (ankyrin repeat domain to rps17) in the ovaries, carcass and eggs of Ae. aegypti in replicate group 2. A Wolbachia densities in the eggs vs the ovaries of Ae. aegypti in replicate group 2. B. Wolbachia densities in the eggs vs the carcass of Ae. aegypti in replicate group 2. n = 18 individuals. [file 13071_2022_5231_MOESM4_ESM.pdf]

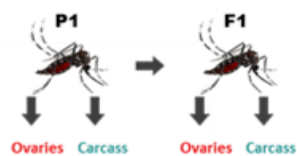

**A**

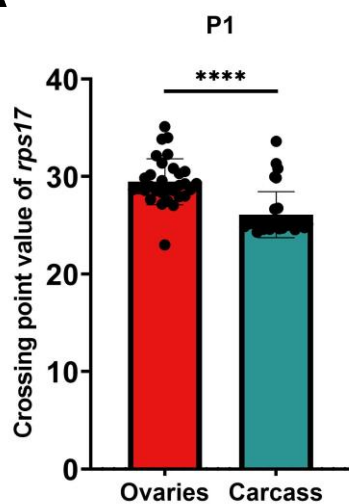

**B**

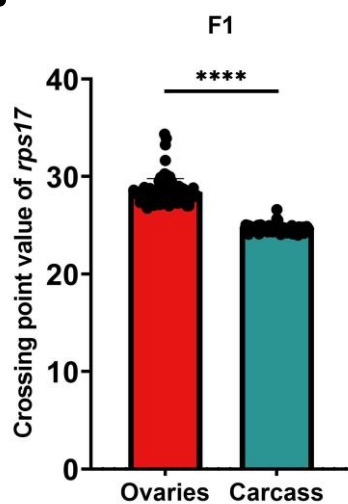

Supplement: Supplementary file 5 — Additional file 5: Figure S5. Crossing point values of rps17 in the tissues of mothers (P1) and daughters (F1). These data pertain to Fig. 6 in the main text. A Crossing point values of rps17 of P1 in Ae. aegypti. B Crossing point values of rps17 of F1 in Ae. aegypti. Figure A has n = 31 individuals while B has n = 78 individuals. Bars indicate tissue means ± SE; ****P ≤ 0.0001. [file 13071_2022_5231_MOESM5_ESM.pdf]

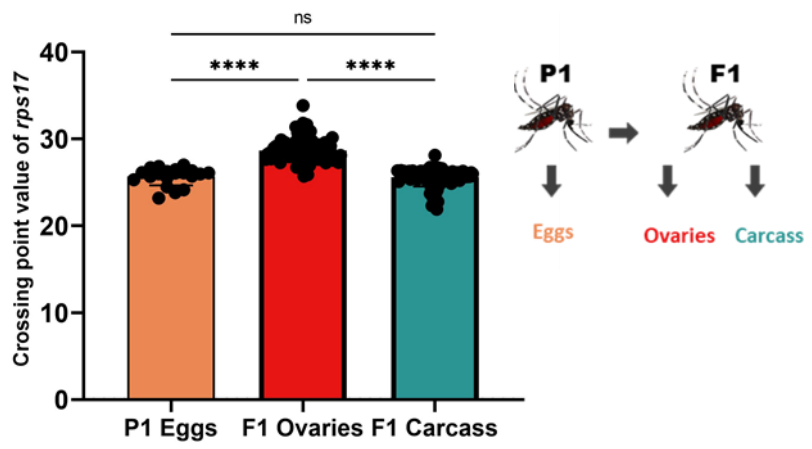

Supplement: Supplementary file 6 — Additional file 6: Figure S6. Crossing point values of rps17 in the eggs of mothers (P1) and tissues of daughters (F1). These data pertain to Fig. 7 in the main text. For P1 eggs n = 20 individuals, while for ovaries and carcass n = 94 individuals. Bars indicate tissue means ± SE. ns, Not significant; asterisks indicate significant difference at ****P ≤ 0.0001. [file 13071_2022_5231_MOESM6_ESM.pdf]

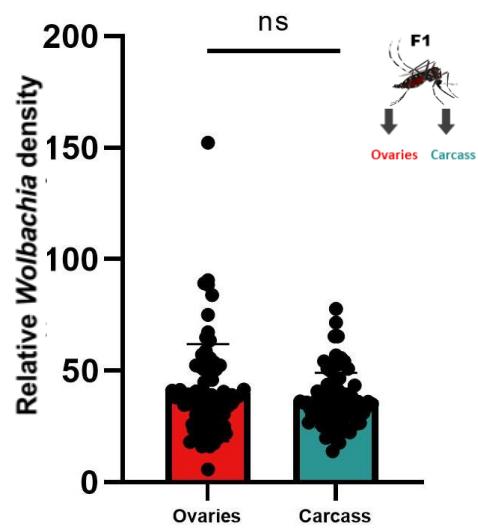

Supplement: Supplementary file 7 — Additional file 7: Figure S7. Relative Wolbachia densities (ankyrin repeat domain to rps17) in the ovaries and the carcass of Ae. aegypti in the same generation of daughters (F1). n = 79, P = 0.21 (paired t-test). Bars indicate tissue means ± SE. ns, Not significant. [file 13071_2022_5231_MOESM7_ESM.pdf]

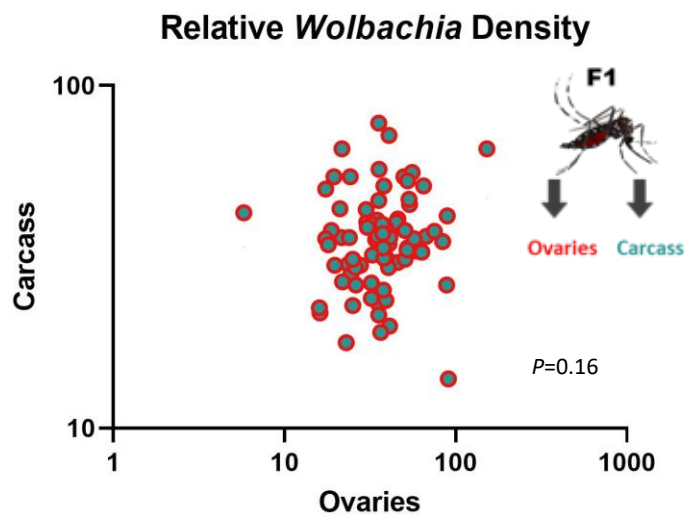

Supplement: Supplementary file 8 — Additional file 8: Figure S8. Relationship between relative Wolbachia densities in the ovaries and the carcass of Ae. aegypti in the same generation of daughters (F1). n = 79. [file 13071_2022_5231_MOESM8_ESM.pdf]
